# Venous thromboembolism prophylaxis in mental healthcare: do the benefits outweigh the risks?

**DOI:** 10.1192/pb.bp.113.046680

**Published:** 2015-04

**Authors:** Rashmi Patel

**Affiliations:** 1 King’s College London, UK

## Abstract

Venous thromboembolism is an important cause of morbidity and mortality. In recent years, growing awareness has led to the development of strategies to prevent venous thromboembolism in individuals admitted to hospital who are deemed to be at high risk. However, there remains a considerable degree of uncertainty over whether these strategies are of overall benefit and there are few published studies on people who are admitted to psychiatric hospitals. In this editorial I review current clinical practice and areas of uncertainty with respect to venous thromboembolism prophylaxis and its implementation in mental healthcare settings.

Previous estimates have suggested that venous thromboembolism is responsible for around 60 000 deaths in the UK each year.^1^ It is thought that individuals admitted to hospital may be at increased risk of developing deep vein thrombosis and/or pulmonary embolism as a result of reduced mobility or intercurrent illness. Other important risk factors include older age (over 60 years), active malignancy, dehydration, inherited or acquired thrombophilia, obesity, previous venous thromboembolism or family history of venous thromboembolism, oral contraceptive pill use, hormone replacement therapy, pregnancy and varicose veins with phlebitis.^2,3^ These risk factors may be applicable to individuals admitted to hospital for medical, surgical or psychiatric care. For these reasons, current clinical guidelines recommend a risk assessment for all people who are admitted to hospital and prescription of a low-dose anticoagulant such as low molecular weight heparin (LMWH) and/or mechanical prophylaxis for those thought to be at high risk.^2^ The decision as to whether to offer venous thromboembolism prophylaxis should be balanced against potential risks, particularly the risk of bleeding with LMWH.

## Evidence base for venous thromboembolism prophylaxis in acute general hospitals

Numerous interventional studies have investigated the role of mechanical and pharmacological prophylaxis in reducing the risk of venous thromboembolism among those admitted to an acute general hospital. Studies have principally focused on patients undergoing orthopaedic surgery, non-orthopaedic surgery or no surgery. Interventional studies have demonstrated a reduction in symptomatic deep vein thrombosis among patients undergoing orthopaedic surgery who receive LMWH (relative risk (RR) = 0.50, 95% CI 0.43–0.59^4^). The use of LMWH in this group is not associated with a significant increase in major bleeding (RR = 0.81, 95% CI 0.38–1.72^4^). Prophylactic LMWH is also associated with a reduction in non-fatal symptomatic venous thromboembolism in patients who are undergoing non-orthopaedic surgery (RR = 0.31, 95% CI 0.12–0.81^5^) and possibly a reduction in symptomatic deep vein thrombosis (RR = 0.47, 95% CI 0.22–1.00^6^) and pulmonary embolism (odds ratio (OR) = 0.70, 95% CI 0.56–0.87^7^) in patients who are not undergoing surgery. However, this is balanced with a significantly increased risk of major bleeding (OR = 1.28, 1.05–1.56^7^). Furthermore, when considering fatal pulmonary embolism or overall mortality, prophylactic LMWH is not associated with significant benefit in any group.^4–7^

## Evidence base for venous thromboembolism prophylaxis in mental healthcare settings

In contrast to studies in acute general hospitals, there is relatively little published evidence investigating venous thromboembolism incidence and the role of pharmacological or mechanical prophylaxis in mental healthcare settings. A recent observational study that included systematic venous ultrasonography identified deep vein thrombosis in 10 out of 449 participants (2.2%) following 10 days of admission to a psychiatric hospital.^8^ A total of 16 out of 458 (3.5%) had experienced an episode of venous thromboembolism by 90 days following admission. Of these, three had a non-fatal pulmonary embolism. The study also showed that venous thromboembolism was more likely in older people (8.6% of those aged over 75 years), which may relate to greater exposure to risk factors such as immobility. Another study based on a review of hospital records revealed 17 confirmed cases of venous thromboembolism among 1495 people (1.1%) admitted to an in-patient mental health service for older people.^9^ This contrasts with an incidence of 2.4% within 91 days among people undergoing total hip arthroplasty surgery^10^ and 1.45 per 1000 person years in the general population.^11^

There is growing evidence from observational studies that suggests a possible association between antipsychotic medications and venous thromboembolism, particularly for clozapine and first-generation antipsychotics.^12^ However, it has been difficult to establish whether this association could be a direct pharmacological consequence of antipsychotics (leading to a prothrombotic state) or if it is mediated through other risk factors that are consequences of antipsychotics, such as obesity or sedation leading to reduced mobility.^13^ Some studies have also pointed towards physical restraint as a potential risk factor for venous thromboembolism in mental healthcare settings.^14–16^

## Areas of uncertainty

A recent meta-analysis has led to increasing controversy over the potential benefits of pharmacological or mechanical measures to prevent venous thromboembolism among hospital patients who are not undergoing surgery.^7^ Although a reduction in non-fatal symptomatic venous thromboembolism was seen with LMWH prophylaxis, this is balanced with an increased risk of bleeding and no overall benefit in terms of reduced mortality. Furthermore, the relative benefit of prophylaxis only translates to a modest reduction in absolute risk; for every 1000 in-patients treated with LMWH, only three cases of pulmonary embolism are prevented balanced with four additional cases of major bleeding.^7^

There is also continued uncertainty about the true incidence of clinically significant venous thromboembolism.^17^ Although data from epidemiological modelling suggests that venous thromboembolism is responsible for around 60 000 deaths each year in the UK,^1^ data from postmortem studies suggest a much lower rate of around 5680 per year.^18^ Whether pharmacological and mechanical prophylaxis could prevent all deaths from venous thromboembolism is also unclear.

## Do people who develop venous thromboembolism always need treatment with anticoagulants?

Some observational studies have employed systematic ultrasound screening to identify asymptomatic as well as symptomatic deep vein thrombosis. Although deep vein thrombosis was identified in 10 out of 449 participants following admission to a psychiatric hospital, seven cases were of distal deep vein thrombosis of which only one case was symptomatic.^8^ The extent to which asymptomatic deep vein thrombosis predisposes an individual to increased risk of mortality remains uncertain, particularly with respect to asymptomatic distal deep vein thrombosis.^19^

The advent of computed tomography pulmonary angiography (CTPA) has led to a substantial increase in the radiological diagnosis of pulmonary embolism.^20^ However, uncertainty is growing over the optimum treatment particularly with respect to whether all those with a radiological diagnosis of pulmonary embolism would benefit from anticoagulation.^21^ It is thought that small subsegmental emboli may not necessarily be associated with adverse clinical outcomes and that the risks of bleeding from treatment with anticoagulants may outweigh any benefits within this group.^22^

## Benefits and risks of venous thromboembolism prophylaxis in mental healthcare settings

There are no published interventional studies that have investigated the potential benefits of venous thromboembolism prophylaxis in mental healthcare in-patient settings. Despite this, there is ongoing interest in developing and utilising risk-screening tools to identify individuals at increased risk of venous thromboembolism for prophylaxis.^23^ Furthermore, there is no published evidence that has investigated the potential harms of venous thromboembolism prophylaxis in this setting. Although risks of bleeding have been well characterised for people admitted to acute general hospitals, it is not clear whether the same risks apply elsewhere. In particular, prolonged use of LMWH can predispose to thrombocytopenia leading to an increased risk of bleeding.^24^ The mean length of stay in an in-patient mental healthcare setting (adult: 52.1 days, older people: 93.2 days) is substantially greater than that of an acute medical unit (5.5 days).^25^ With the exception of those taking clozapine, full blood count monitoring is not routinely performed in the mental healthcare in-patient setting. The extent to which staff in mental healthcare settings are trained to administer prophylaxis and recognise potential adverse complications is also unclear.^3^ For these reasons, it is possible that the risk of thrombocytopenia from LMWH may be greater for those who receive it for venous thromboembolism prophylaxis in the mental healthcare setting.

Balancing the potential risks of bleeding and the potential benefits of preventing venous thromboembolism with pharmacological prophylaxis is problematic. Cost–utility analysis is a method by which the benefits and risks of an intervention may be balanced with respect to quality of life measures. A study investigating the application of cost–utility analysis to venous thromboembolism found that there was a wide degree of variation in individual estimates of cost–utility of both acute venous thromboembolism and bleeding complications from pharmacological prophylaxis.^26^ However, in the mental healthcare in-patient setting, it is sometimes not possible for patients to weigh up benefits and risks of an intervention because of lack of mental capacity. Furthermore, there is little evidence to estimate the potential benefits and risks of venous thromboembolism prophylaxis among individuals who lack capacity as randomised controlled trials have excluded these individuals.^17^

## Discussion

Venous thromboembolism remains an important cause of mortality in people who are admitted to hospital. However, in recent years, there has been ongoing uncertainty over the efficacy and risks of prophylaxis among in-patients who are not undergoing surgery^6,7^ and whether everyone with established venous thromboembolism would benefit from anticoagulant treatment.^21,22^ Although prophylaxis appears to reduce the incidence of non-fatal venous thromboembolism, there is no robust evidence that supports a reduction in mortality.^4–7^ This may be because of the balance with risk of bleeding for pharmacological prophylaxis.^7,24^ There is even less evidence to support its use in mental healthcare in-patient settings where no interventional studies have been published.

Despite this, substantial resources (over £30 million per year in England) have been invested into venous thromboembolism prevention programmes that claim to ‘save lives’.^27^ Although it is claimed these investments have resulted in a modest overall saving (a yield of 2.7%^28^), it is possible that there is a greater opportunity cost in mental healthcare settings where there is currently no evidence for the cost-effectiveness of venous thromboembolism prophylaxis.

It is clear that there is an ongoing need to improve the overall physical health of individuals with mental illness, particularly those with severe mental illness who have been shown to have a substantially lower life expectancy than the general population.^29^ Although venous thromboembolism is an important cause of mortality, a greater degree of impact could be achieved by investing resources into improving detection and treatment of new cases^3^ as well as preventative strategies in mental healthcare for cardiovascular disease in general.^30^ In summary, there is little evidence to support current strategies for venous thromboembolism prophylaxis in mental healthcare settings. Further study to develop and evaluate the effectiveness of novel venous thromboembolism prevention and early detection strategies is therefore warranted.
